# Association of Weekly Suicide Rates with Temperature Anomalies in Two Different Climate Types

**DOI:** 10.3390/ijerph111111627

**Published:** 2014-11-13

**Authors:** P. Grady Dixon, Mark Sinyor, Ayal Schaffer, Anthony Levitt, Christa R. Haney, Kelsey N. Ellis, Scott C. Sheridan

**Affiliations:** 1 Department of Geosciences, Fort Hays State University, Hays, KS 67601, USA; 2 Sunnybrook Health Sciences Centre, Department of Psychiatry, University of Toronto, Toronto, ON M4N 3M5, Canada; E-Mails: mark.sinyor@sunnybrook.ca (M.S.); ayal.schaffer@sunnybrook.ca (A.S.); anthony.levitt@sunnybrook.ca (A.L.); 3 Department of Geosciences, Mississippi State University, Mississippi State, MS 39762, USA; E-Mail: meloche@geosci.msstate.edu; 4 Department of Geography, University of Tennessee, Knoxville, TN 37996, USA; E-Mail: ellis@utk.edu; 5 Department of Geography, Kent State University, Kent, OH 44242, USA; E-Mail: ssherid1@kent.edu

**Keywords:** suicide, climate, meteorology, Canada, Mississippi, temperature, weather

## Abstract

Annual suicide deaths outnumber the total deaths from homicide and war combined. Suicide is a complex behavioral endpoint, and a simple cause-and-effect model seems highly unlikely, but relationships with weather could yield important insight into the biopsychosocial mechanisms involved in suicide deaths. This study has been designed to test for a relationship between air temperature and suicide frequency that is consistent enough to offer some predictive abilities. Weekly suicide death totals and anomalies from Toronto, Ontario, Canada (1986–2009) and Jackson, Mississippi, USA (1980–2006) are analyzed for relationships by using temperature anomaly data and a distributed lag nonlinear model. For both analysis methods, anomalously cool weeks show low probabilities of experiencing high-end suicide totals while warmer weeks are more likely to experience high-end suicide totals. This result is consistent for Toronto and Jackson. Weekly suicide totals demonstrate a sufficient association with temperature anomalies to allow some prediction of weeks with or without increased suicide frequency. While this finding alone is unlikely to have immediate clinical implications, these results are an important step toward clarifying the biopsychosocial mechanisms of suicidal behavior through a more nuanced understanding of the relationship between temperature and suicide.

## 1. Introduction

According to the World Health Organization [1], global annual suicide numbers are greater than the total deaths from homicide and war combined, despite known underreporting in most countries [2,3,4]. Such a statistic is a bit ambiguous due to the definition of the category of “war,” but the broad point is perfectly clear: suicide is one of the most-common preventable causes of death in the world. Since 2009, suicide deaths have exceeded deaths by car accidents in the United States [5,6]. In addition to emotional impacts, suicide deaths exact socioeconomic tolls due to increased health care costs, legal expenses, missed work, and lost productivity by surviving friends and relatives [7]. 

Suicide is a complex behavioral endpoint, and there are many biopsychosocial and environmental factors that influence the risk of suicide and resulting population [8]. Hence, a simple cause-and-effect model with weather variables such as temperature seems highly unlikely. Nevertheless, careful study of how, if at all, the weather may impact frequencies of suicide could yield important insight into the biopsyhcosocial mechanisms involved in suicide deaths. Deisenhammer [9] provides an extensive review of previous weather-suicide research results while also explaining that, despite much research on the topic, relationships between weather and suicide are still not well characterized. Previous studies have produced mixed results with some showing no significant relationships between weather and suicide deaths [9,10,11,12,13,14,15,16,17], some finding suicide deaths are more likely during colder weather [18,19,20,21,22], some finding suicide deaths are more likely during warmer weather [9,23,24,25,26,27,28,29,30,31,32,33], and some finding relationships with weather variables other than temperature [34,35,36,37,38,39]. In a review paper focused primarily on the shortcomings and likely causes for inconsistent findings among previous studies, Dixon and Kalkstein [40] suggest longer study periods, use of multi-day data with lag considerations, careful data normalization, more collaborative efforts between health professionals and geographer-climatologists, and increased comparisons across political and cultural boundaries. 

Several of the recommendations listed by Dixon and Kalkstein [40] can be addressed efficiently through the use of a distributed lag nonlinear model (DLNM), which has been growing in popularity among those that study environmental effects on human health and mortality [41,42,43,44,45]. This recently developed statistical model is designed to estimate simultaneously the nonlinear and cumulative lag effects of exposure to an independent exposure variable (*i.e*., temperature, pollution, humidity, etc.) on mortality or morbidity. The flexibility and efficiency of combining the relationship in terms of the exposure variable as well as the dimension of delayed effects is well-suited for research in environmental epidemiology. However, the “cross-basis” function allows independent functions for the exposure variable and the lag, so one can be used to assess the strongest signals with respect to the other. Further, the model readily allows for normalization with respect to time at user-defined scales to control for periodic patterns (*i.e*., weekly or seasonal) and long-term trends. The purposes of the current paper are to follow the suggestions of Dixon and Kalkstein [40] to help improve the methods used by those studying weather-suicide relationships while also providing more robust analyses of the commonly studied, but still ambiguous, association between air temperature and suicides.

## 2. Materials and Methods

### 2.1. Suicide Data

**Figure 1:**
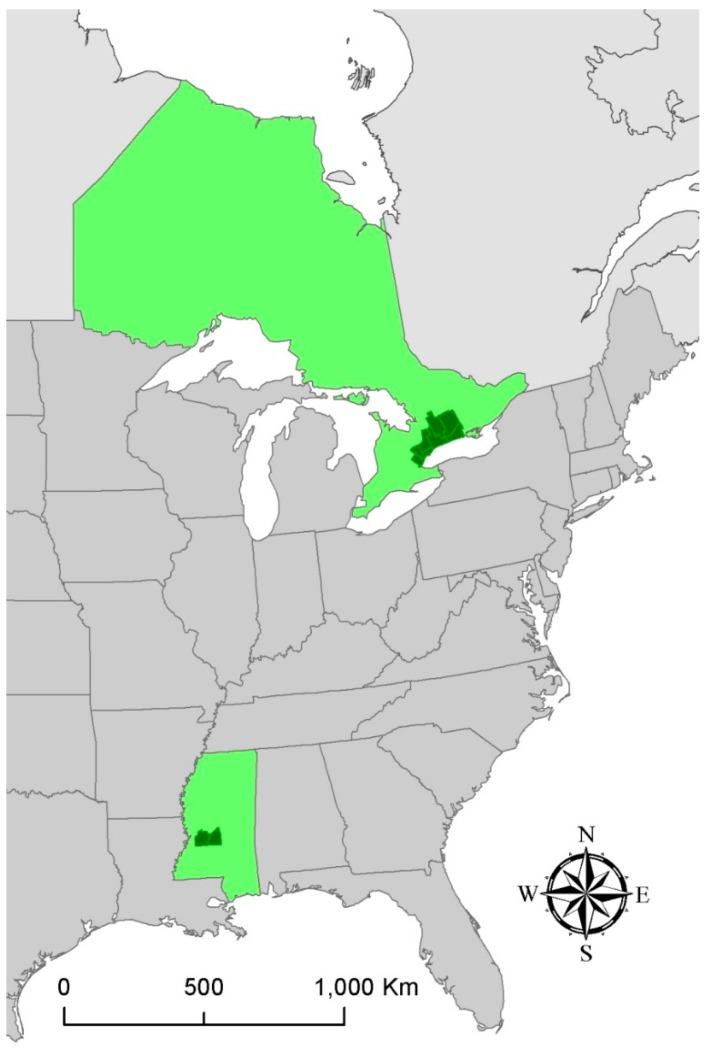
Study sites. Ontario and Mississippi are highlighted in light green while the Toronto and Jackson metropolitan areas are in dark green.

Suicide data from the Toronto Metropolitan Area (Figure 1) during 1986–2009 were provided by the Office of the Chief Coroner of Ontario. Each record includes date, sex, age, and municipality of the suicide. There are 10,772 records for the entire study period, and annual suicide totals ranged from 401 in 1999 to 502 in 2003 with a median of 441. Males make up 72% of the records. Analysis by age shows that 36% were at least 50 years old, 33% were ages 35–49, and 24% were ages 22–34. 

Data from Mississippi (Figure 1) include all suicides in Hinds and Rankin Counties (two of the three metropolitan counties for the city of Jackson) for the years 1980–2006. These were provided by the Mississippi State Department of Health, and each record includes date, sex, age, and county of suicide. Data from Madison County, the third county in the Jackson Metropolitan Area, are not available at the level of detail as the others because of its smaller population (*i.e*., <100,000). There are 989 records for the entire study period, and annual suicide totals ranged from 25 in 1981 to 51 in 1993 with a median of 36. Males make up 77% of the records. Analysis by age shows that 32% were at least 50 years old, 26% were ages 35–49, and 28% were ages 22–34.

According to the United States Department of Health and Human Services (http://www.hhs.gov/ohrp/policy/engage08.html), “*human subject”* is defined as a living individual about whom an investigator conducting research obtains:

1. data through intervention or interaction with the individual, or
2. identifiable private information.

This project does not meet the regulatory definition of “human subjects research” because it does not include collection of information about living individuals. This was confirmed by an Institutional Review Board expert in the Office of Regulatory Compliance at Mississippi State University (HRPP Study #M2013-028 NHSR).

### 2.2. Meteorological Data

Daily surface observational data for Toronto Pearson International Airport (YYZ) in Toronto, Ontario (43.68°N latitude) for the years 1986–2009 were obtained from Environment Canada. Comparable data for Hawkins Field Airport (KHKS) in Jackson, Mississippi (32.33°N latitude) for the years 1980–2006 were obtained from the National Climatic Data Center. Data include daily maximum and daily minimum temperatures. Daily maximum temperatures for Toronto ranged from −21.0 °C to 37.9 °C with an average of 13.2 °C while the daily minimum temperatures ranged from −31.0 °C to 26.3 °C with an average of 3.6 °C. Conversely, daily maximum temperatures for Jackson ranged from −7.8 °C to 41.7 °C with an average of 24.6 °C while the daily minimum temperatures ranged from −16.7 °C to 26.7 °C with an average of 12.0 °C.

### 2.3. Weekly Anomalies

To improve the chances of identifying a reliable relationship between temperature and suicides, each daily suicide total was compared to the average value for that day of the year resulting in a calculated daily anomaly. Daily anomalies were summed over seven-day periods to assess weekly anomalies. Weeks with the greatest negative and positive departures were used to identify “high-end” and “low-end” suicide weeks. The 5th and 95th percentiles were used for this, which resulted in high-end events (greater than or equal to the 95th percentile) being selected if they experience a weekly anomaly of at least 5.0 while low-end events (less than or equal to the 5th percentile) experienced anomalies equal to or less than −4.5.

It is expected that temperature values during high-end events will vary from those during low-end events, but this may not be evident in all months. Therefore, daily anomalies were calculated for maximum and minimum diurnal temperatures, and these anomalies were averaged over the preceding seven days. Monthly averages of the seven-day temperature (daily maximum and minimum) anomalies were calculated for high-end and low-end suicide weeks. The purpose of this analysis is to highlight months that show the greatest temperature differences between high-end and low-end suicide weeks.

The months with the greatest temperature differences between high-end and low-end suicide weeks were selected for further analysis. The weekly suicide anomalies were then plotted against the weekly temperature anomalies (maximum and minimum) for the selected months. A third temperature category was calculated by selecting the greatest absolute value of weekly anomaly (either maximum or minimum daily temperatures). This “greatest departure” variable accounts for the possibility that suicide counts are affected by any dramatic departure in temperature rather than for maximum or minimum values, specifically.

### 2.4. Distributed Lag Nonlinear Model

After initial analyses of the observational data provided some insight into the temperature-suicide relationships, we used the “dlnm” package in R (http://cran.r-project.org/web/packages/dlnm/) to analyze the relationship using all available temperature data (rather than just the extremes). We used the general linear model regression function to use daily temperature values (maximum and minimum) to create estimations of daily suicide counts. Natural cubic splines allow for normalization with respect to various time scales. We compiled the model for annual, biannual, and seasonal scales, so we allowed for equally spaced spline knots totaling 11, 5, and 3, respectively. Seasons were defined as winter (Dec–Feb), spring (Mar–May), summer (Jun–Aug), and fall (Sep–Nov), and the “warm season” was defined as Mar–Aug to capture the frequent suicide peak during late spring and summer. We also employed a categorical “day of week” spline to account for weekly cycles/patterns. Consistent with Rocklov *et al.* [42], we allowed the temperature spline to have 3–8 degrees of freedom. We tested lag times of up to one week (*i.e*., a 6-day lag with “day 0” representing the first exposure day), and we allowed the lag polynomial function to have 2–4 degrees of freedom. Akaike Information Criterion (AIC) values were used to conduct a sensitivity analysis and ultimately choose the appropriate degrees of freedom for each iteration of the DLNM using data from Toronto. The relative risk of suicide mortality due to the various temperature exposures were compared to the location’s median temperature value for the period in question.

## 3. Results

### 3.1. Monthly Average Anomaly Differences for Toronto

**Figure 2:**
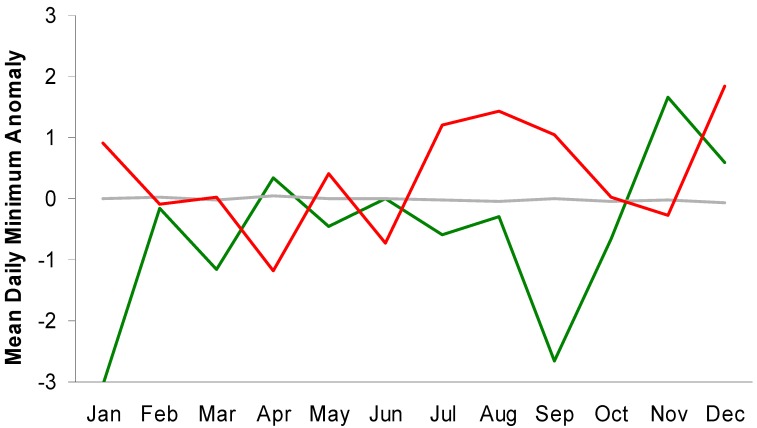
Monthly values for mean 7-day anomalies of minimum daily temperatures for high-end (red), low-end (green), and all other (gray) suicide weeks in Toronto.

Four months of the year (Jan, Jul, Aug, and Sep) show much larger differences in the seven-day mean minimum daily temperature anomalies for high-end versus low-end suicide weeks (Figure 2). 

**Figure 3:**
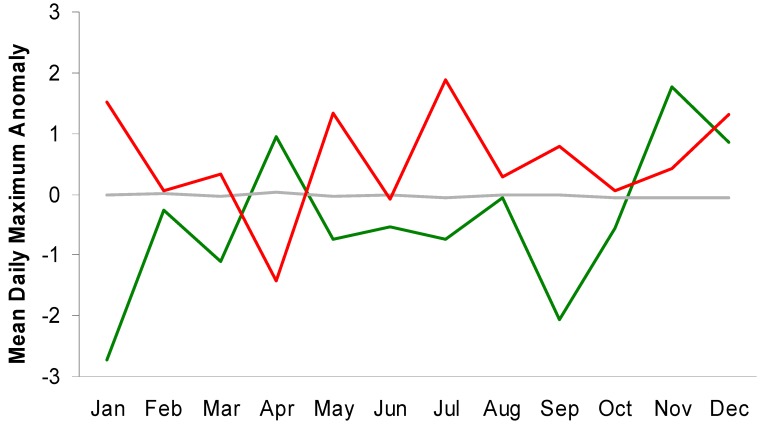
Monthly values for mean 7-day anomalies of maximum daily temperatures for high-end (red), low-end (green), and all other (gray) suicide weeks in Toronto.

A similar, though less consistent pattern is evident for maximum daily temperatures (Figure 3). These results suggest that attempts to use temperature to separate weeks with relatively high or low suicide totals may be more successful by focusing on certain times of the year. This is particularly relevant simply because many locations, including Toronto, experience their greatest suicide counts during the warmest months.

### 3.2. Toronto Scatterplot Anomaly Categories

**Figure 4:**
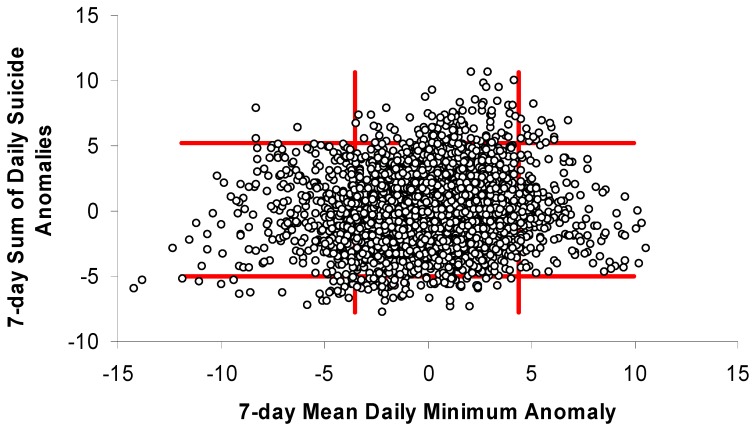
Weekly suicide anomalies plotted against weekly anomalies of daily minimum temperature for selected months (Jan, Jul, Aug, Sep) in Toronto. Red bars help to identify temperature-anomaly thresholds that may improve prediction of suicide anomalies.

**Figure 5:**
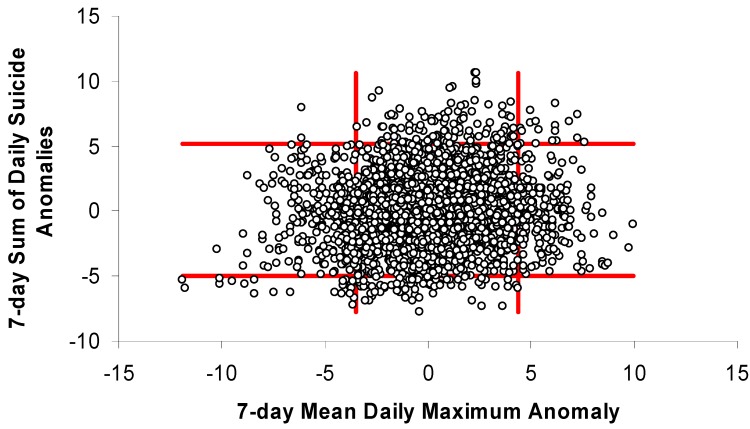
Weekly suicide anomalies plotted against weekly anomalies of daily maximum temperature for selected months (Jan, Jul, Aug, Sep) in Toronto. Red bars help to identify temperature-anomaly thresholds that may improve prediction of suicide anomalies.

**Figure 6:**
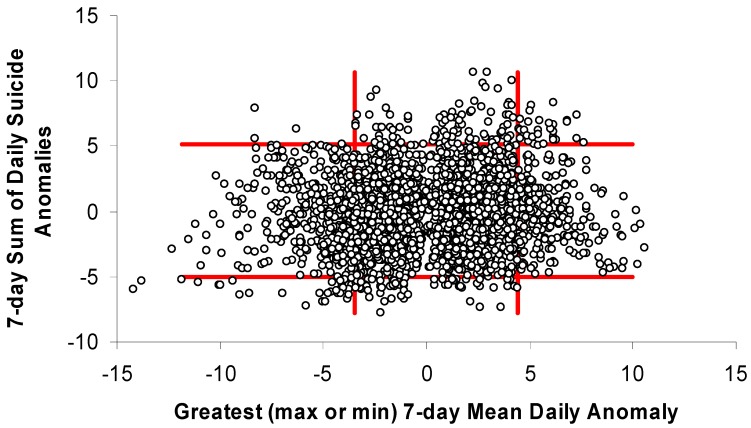
Weekly suicide anomalies plotted against weekly anomalies of greatest absolute daily departures from climatological normal temperature (maximum or minimum) for selected months (Jan, Jul, Aug, Sep) in Toronto. Red bars help to identify temperature-anomaly thresholds that may improve prediction of suicide anomalies.

The months selected for having large differences in temperatures associated with high-end and low-end suicide weeks (Jan, July, Aug, and Sep) in Toronto were analyzed with simple scatterplots (Figure 4, Figure 5 and Figure 6). While the scatterplots do not show strong universal relationships throughout the distribution, they do suggest that weeks with the greatest positive (negative) suicide anomalies almost never occur during weeks that are much cooler (warmer) than normal. The temperature-anomaly and suicide-anomaly categories used for this analysis were chosen to maximize the chances of successfully predicting weeks with or without high or low suicide anomalies. This resulted in ~24% of days being captured by the cool and warm categories (cool = 14%; warm = 10%) and the remaining values being considered “normal.” When using the greatest daily temperature anomaly to classify weeks as “cool,” “normal,” or “warm,” none of the warm weeks experienced low suicide anomalies while nearly 10% saw high suicide anomalies (Table 1). Similarly, cool weeks experienced high and low suicide anomalies 0.7% and 7.4% of the time, respectively.

**Table 1:** Summary of the frequency of days for each combination of suicide and temperature anomalies for Toronto.

Suicide Anomalies	Daily Minimum Temperatures				Daily Maximum Temperatures				Greatest Absolute Temperature Anomaly		
	Cool	“Normal”	Warm		Cool	“Normal”	Warm		Cool	“Normal”	Warm
Total Days for Each Combination of Suicide and Temperature Anomalies											
High	3	98	18		2	102	15		3	89	27
“Normal”	326	2183	222		255	2300	176		387	2089	255
Low	27	69	0		22	74	0		31	65	0
Combinations as Percentages of Days in Each Temperature Anomaly Category											
High	0.8	4.2	7.5		0.7	4.1	7.9		0.7	4.0	9.6
“Normal”	91.6	92.9	92.5		91.4	92.9	92.1		91.9	93.1	90.4
Low	7.6	2.9	0.0		7.9	3.0	0.0		7.4	2.9	0.0

### 3.3. Jackson, Mississippi Comparison

**Figure 7:**
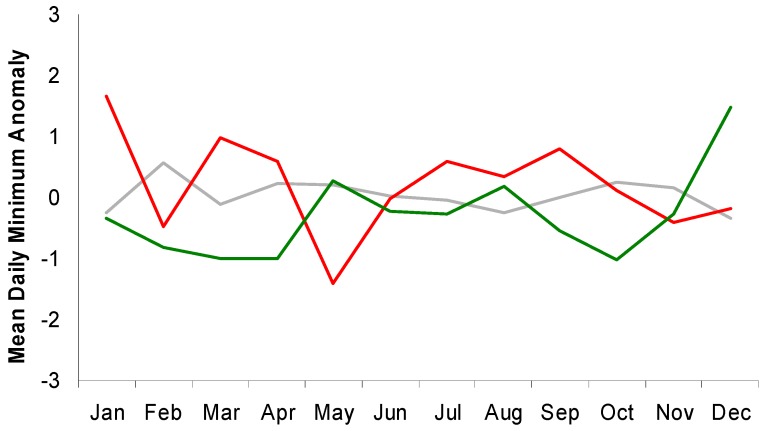
Monthly values for mean 7-day anomalies of minimum daily temperatures for high-end (red), low-end (green), and all other (gray) suicide weeks in Jackson, Mississippi.

Results of the previous analysis can be interpreted to mean that warmer-than-average weeks are associated with higher-than-normal suicide totals during certain months of the year. In Canada, warmer conditions may evoke different physical, emotional, and behavioral responses than in other locations. So, to better understand the relationship, and to determine if it exists outside of Toronto, the same analysis was performed for Jackson, Mississippi (Figure 6) because it regularly experiences mild winters and longer, hotter summers. Monthly differences in average temperature anomalies for high-end and low-end suicide weeks show some similar, although not identical, patterns as those found for Toronto (Figure 7). This is partially explained by the much smaller population and, therefore, fewer daily suicides in Jackson as this makes it difficult for days to experience large negative anomalies during some months. Therefore, the temperature anomalies for high-end suicide weeks should perhaps be compared to all other weeks rather than just the low-end events. Accordingly, it can be seen that the same months (Jan, Jul, Aug, Sep) used in the Toronto analysis tend to experience relatively large differences. The months of March and April also show large differences in temperature anomalies for high-end and low-end suicide events, but the rest of this comparison uses the same months as Toronto for the sake of consistency.

**Figure 8:**
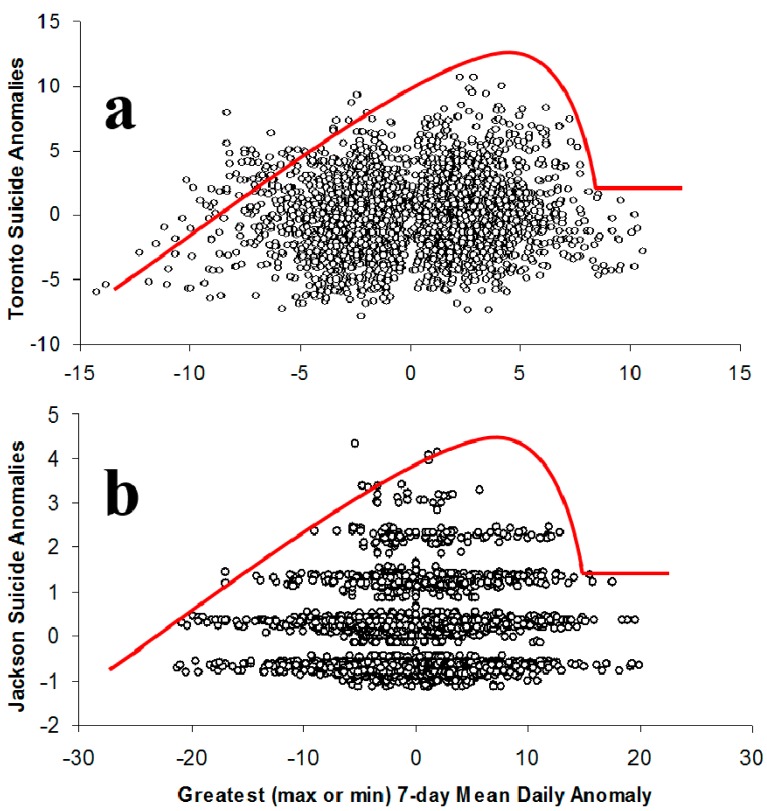
Comparison of the suicide-temperature anomaly scatterplots for Toronto (**a**) and Jackson (**b**). The red curve illustrates the similar absence of high-suicide weeks during cooler conditions and the warmest conditions.

A comparison of scatterplots from Jackson and Toronto reveals similar relationships between temperature and suicide anomalies (Figure 8). Specifically, both locations show a lack of high-end suicide weeks when temperatures are cooler-than-normal. Both locations experience an increase in high-end suicide weeks as the temperatures warm, but that trend reverses for the warmest (*i.e*., the top 1%) weeks as no high-end suicide weeks occur for these periods in either location. 

### 3.4. DLNM Results

**Figure 9:**
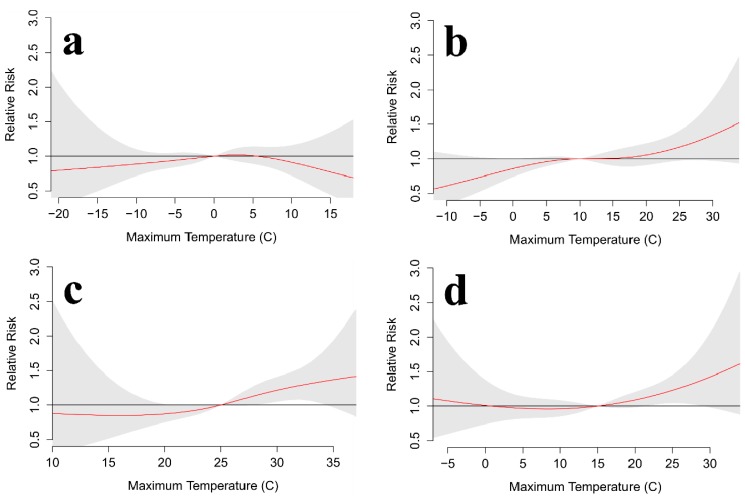
Toronto relative risk for suicide at various daily maximum temperatures over a 6-day lag for (**a**) winter, (**b**) spring, (**c**) summer, and (**d**) fall. The red line is the predicted value and the gray shading represents the 95% confidence interval.

**Figure 10:**
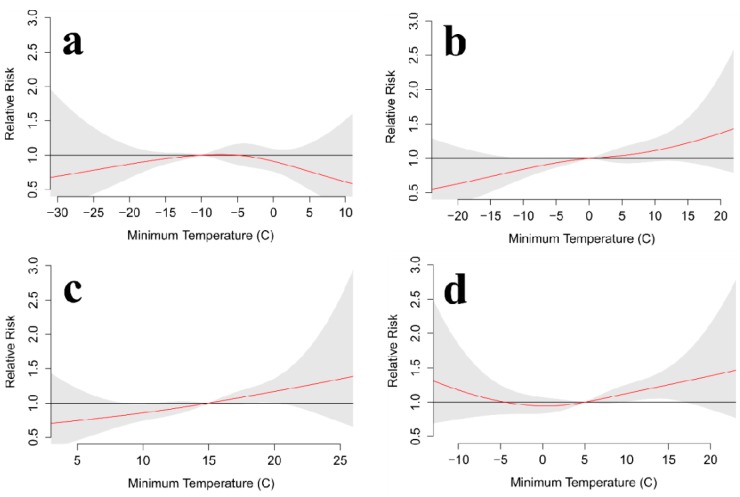
Toronto relative risk for suicide at various daily minimum temperatures over a 6-day lag for (**a**) winter, (**b**) spring, (**c**) summer, and (**d**) fall. The red line is the predicted value and the gray shading represents the 95% confidence interval.

AIC values suggest that a 6-day lag, along with fewer degrees of freedom for the temperature spline (3–4) and the lag polynomial (2–3), provides the best balance of model fit and complexity (Table 2). Seasonal analyses for Toronto show some consistency as results for spring, summer, and fall each suggest that suicides are more likely during warmer-than-normal conditions (Figure 9 and Figure 10). Spring is the only season to show a clear decrease in suicides under cooler-than-normal conditions for daily maximum and minimum temperatures, but summer minimum temperatures show a very similar pattern. Winter differs from the rest as it shows that suicides tend to decrease for both anomalously warm and cool temperatures.

**Table 2:** AIC values for the DLNM using Toronto maximum daily temperatures with various lag durations (3–6 days), lag polynomial function degrees of freedom (2–4), and temperature spline degrees of freedom (3–8).

Lag Poly	3-Day Lag		4-Day Lag		5-Day Lag		6-Day Lag	
*df*	Temperature Spline *df*	AIC	Temperature Spline *df*	AIC	Temperature Spline *df*	AIC	Temperature Spline *df*	AIC
2	3	24,742	3	24,655	3	24,582	3	24,515
2	4	24,743	4	24,657	4	24,585	4	24,514
2	5	24,747	5	24,662	5	24,589	5	24,518
2	6	24,749	6	24,667	6	24,593	6	24,522
2	7	24,754	7	24,671	7	24,599	7	24,526
2	8	24,756	8	24,676	8	24,601	8	24,528
3	3	24,746	3	24,660	3	24,588	3	24,521
3	4	24,748	4	24,659	4	24,592	4	24,522
3	5	24,753	5	24,666	5	24,595	5	24,529
3	6	24,751	6	24,672	6	24,602	6	24,534
3	7	24,755	7	24,676	7	24,610	7	24,539
3	8	24,757	8	24,679	8	24,611	8	24,544
4	na	na	3	24,665	3	24,592	3	24,527
4	na	na	4	24,666	4	24,594	4	24,525
4	na	na	5	24,673	5	24,598	5	24,532
4	na	na	6	24,672	6	24,607	6	24,539
4	na	na	7	24,675	7	24,612	7	24,546
4	na	na	8	24,679	8	24,614	8	24,549

**Figure 11:**
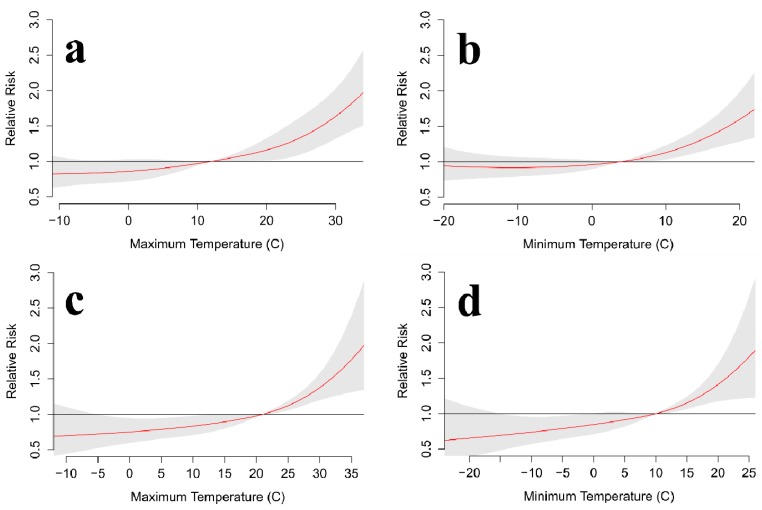
Toronto relative risk for suicide at various daily (**a**,**c**) maximum and (**b**,**d**) minimum temperatures over a 6-day lag for (a,b) the entire year and (c,d) the warm season (March–August). The red line is the predicted value and the gray shading represents the 95% confidence interval.

Sample size is always a concern with analysis of daily suicide totals, so it is not surprising to see smoother modeled relationships for longer periods that are less affected by individual days (Figure 11). For both daily maximum and minimum temperatures, the annual association between temperature and suicide suggests that suicides are more likely during warmer conditions. Temperatures below the median are slightly less likely to see suicide events, but the relative risk of warmer temperatures rises to approximately twice that of the median temperature. Perhaps more importantly, the narrower confidence intervals allow for the identification of statistically significant differences in the suicide risk for temperatures below the median and those above the median. These statistically significant differences are also present for every few degrees of warming at the warmest temperatures.

**Figure 12:**
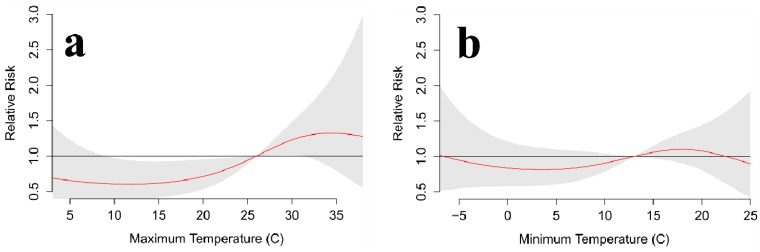
Jackson, MS annual relative risk for suicide at various daily (**a**) maximum and (**b**) minimum temperatures over a 6-day lag. The red line is the predicted value and the gray shading represents the 95% confidence interval.

DLNM results for Jackson, Mississippi are similar to those for Toronto, but the relationship does not appear as reliable. This could be due to the much smaller sample size, which leads to more noise in the model. Nevertheless, it appears still that warmer-than-normal temperatures yield a statistically significant increase in suicides over most cooler-than-normal temperatures, especially for daily maximum temperatures (Figure 12). For daily minimum temperatures, the pattern is similar, but there are less-amplified differences between suicide totals for warmer and cooler conditions. Biannual and seasonal analyses produced noisy results for Jackson, likely due to sample size, so those are not shown.

## 4. Discussion

Thus far, research on the effects of weather on suicide has been largely ambiguous. Seasonality is a consistent finding [46,47,48,49,50], and most studies report some noteworthy associations between suicide counts and at least one weather variable [9]. While the associations in the current study may not help us specifically to predict or prevent suicide deaths, they clarify and significantly advance our understanding of the relationships between suicide and weather, particularly temperature. The methods of analyzing anomalies for specific times of year and focusing on the periods and events showing the strongest signals are instructive for future researchers. The use of the DLNM may also encourage researchers to seek methods developed and used outside of their discipline, while use of multiple datasets from significantly different locations (geographically, climatically, politically, *etc.*) may encourage future researchers to confirm their results similarly. 

According to analyses relying solely on the observational data as well as the DLNM statistical model, high-end suicide anomalies are unlikely in cooler weather and low-end suicide anomalies are unlikely in warm weather. It seems even that warmer-than-normal temperatures are associated with increasing suicide totals during much of the year. The consistency of these results between seasons and in both locations (Toronto and Jackson) indicates that temperature may have a meaningful, albeit perhaps small, effect on suicide deaths. It should be noted that Jackson lacks a pronounced group of low-end suicide anomalies because so many days in that city experience zero suicides due primarily to its lower population. This makes it difficult to achieve large negative anomalies, but the similarities between the two cities, despite this difference (and many others), enhances the confidence in the results of this study.

## 5. Conclusions

This study yields a more nuanced and robust understanding of the relationship between suicide and temperature, and it encourages more rigorous testing of analytical results by using data from multiple, distinct locations. Ultimately, one may be able to predict weeks with high probabilities of suicide anomalies to allow more focused prevention efforts. That seems implausible at present, but this study has the potential to advance our understanding of the relationship between weather and suicide while illustrating that weather-suicide relationships are likely strong enough to consider predictive strategies as a possibility. Future research should move beyond asking whether there are systematic relationships between suicides and weather. Instead, focus must be placed on understanding the relationships and using them to predict changes in suicide frequency at various temporal and spatial scales.

This project should be viewed as an important step in addressing the ambiguity in the existing literature on weather-suicide relationships. It is anticipated that future studies will build upon these methods as researchers probe the relationships between weather and suicide. In addition to temperature, research on weather parameters such as sunlight, barometric pressure, and air quality is likely to benefit from methods similar to those used here. With sufficient suicide data, analyses can also be separated according to demographics and/or method of suicide as done by Räsänen *et al.* [50], which shows differences in seasonality for various methods of suicide. A logical next step in the research will be to determine if certain types of suicides are affected by short-term weather changes similarly to violent or aggressive behaviors.
